# Supplementary material for: Relationships among Indicators of Metabolism, Mammary Health and the Microbiomes of Periparturient Holstein Cows
Source: Animals (Basel). 2021 Dec 21;12(1):3. doi: 10.3390/ani12010003 (PMC8749929; doi:10.3390/ani12010003)

Supplementary Material

Table S1. Diet A provided to dams between 60 and 30 days and diet B provided to dams between 30d to calving time during the experiment.

Diet A (60-30d from expected calving)		Diet B (30d to calving)	
**Item**	**% DM**	**Item**	**% DM**
Concentrate A	5.92	Anionic concentrate	9.06
Soy bran	8.33	Wheat straw	12.41
Wheat straw	7.80	Soybean meal	19.82
Tifton grass	13.25	Corn silage	58.71
Corn silage	64.70	-	
Total	100	Total	100

Table S2. Concentrate A provided to dams between 60 and 30 days and Anionic concentrate provided to dams between 30 days to calving time during the experiment (recipe to 1000.9 kg).

Concentrate A		Anionic concentrate	
**Item**	**Quantities (Kg)**	**Item**	**Quantities (Kg)**
Vitamin E	2	NutriCAB®	25
Corn grain	1086	Colina	15
570 ca (Nutron)	510	Vitamin E	1
Urea	200	Salus NA	440
OmniGen® (Phibro)	100	Corn grain	425
Yeast	50	Vitamin AD3	250
Magnesium oxide	25	OmniGen® (Phibro)	40
Mycofix® (Biomin)	20	Yeast	25
Sel-plex® (Alltech)	2	Urea	15
Difly® (Champion)	1.5	Mycofix® (Biomin)	7
Biotin	1.0	Nexulin® (Pancosma)	41
-	-	Biotin	1
-	-	Sel-plex® (Alltech)	1
-	-	Difly® (Champion)	1
-	-	Probios® (Ourofino Saúde Animal)	0.5

Table S3. Distribution of the clinical signals obtained by semiological techniques according to different categories and score adopted to the mammary gland and colostrum examination immediately after calving.

Semiological Techniques	Categories	Score
	General volume normal	0
	General volume increased	1
	General volume diminished	2
	No localized volume	0
Inspection of mammary gland	Localized volume present	1
	Normal coloration	0
	Reddish coloration	1
	Purplish coloration	2
	Soft consistency	0
	Soft with small nodules	1
	Soft consistency with hard nodules	2
	Firm consistency	3
Palpation of mammary gland	Edemaciated	4
	Diffuse firm consistency	5
	Normal temperature	0
	Increased temperature	1
	Normal skin elasticity	0
	Diminished skin elasticity	1
Palpation of Lymph nodes	Normal volume	0

	Increased volume	1
	No alteration in secretion	0
	Watery secretion	1
Strip cup	Watery secretion and presence of clots	2
	Presence of clots, flakes, or other abnormal secretion	3
	No precipitate	0
	Distinct precipitate/weak gel formation	1
California Mastitis Test	Distinct gel formation	2
	strong gel formation	3

Table S4. P values of the Spearman correlations between health parameters and the colostrum microbiome of Holstein cows at calving time.

	P values										
**Taxa classification family - genus**	**Colostrum volume**	**IgG Elisa**	**NEFA**	**Total protein**	**Albumin**	**Triglycerides**	**Cholesterol**	**Time in dry period**	**Macro-phages/epi-thelial cells**	**Score MG**	**Milk pro-duction**
Rhodocyclaceae Thauera	0.702	0.706	0.053	0.335	0.229	0.011*	0.520	0.381	0.273	0.235	0.106
Bacillaceae Bacillus	0.405	0.638	0.073	0.235	0.471	0.005*	0.785	0.511	0.232	0.061	0.061
Rhizobiaceae Mesorhizobium	0.624	0.289	0.135	0.077	0.519	0.029*	0.972	0.777	0.082	0.986	0.236
Bacillaceae Bacillus	0.658	0.596	0.877	0.142	0.591	0.008*	0.906	0.703	0.084	0.144	0.063
Rhodothermaceae uncultured	0.651	0.561	0.115	0.186	0.105	0.499	0.006*	0.836	0.856	0.060	0.230
uncultured	0.557	0.329	0.899	0.475	0.184	0.394	0.010*	0.498	0.244	0.071	0.063
Peptostreptococcaceae uncultured	0.885	0.449	0.189	0.595	0.253	0.639	0.255	0.037*	0.039*	0.822	0.071
Pseudomonadaceae Pseudomonas	0.740	0.338	0.780	0.126	0.237	0.055	0.014*	0.952	0.024*	0.485	0.356
Pseudomonadaceae Pseudomonas	0.523	0.444	0.789	0.566	0.493	0.106	0.051	0.008*	0.019*	0.872	0.013*

*P values > 0.05 were considered significant.

Table S5. P values of the Spearman correlations between health parameters and the fecal microbiome of Holstein cows at calving time.

	P values										
**Taxa classification family - genus**	**NEFA**	**BHB**	**Total pro-tein**	**Albumin**	**Triglycerides**	**Cholesterol**	**Hp**	**Milk pro-duction**	**Last lactation time**	**BSC**	**Weight**
Peptostreptococcaceae Romboutsia	0.614	0.529	*0.016	0.150	*0.020	*0.026	*0.016	0.189	*0.001	0.892	0.886
Bacteroidaceae Bacteroides	0.011	*0.004	0.402	0.625	0.290	0.525	0.124	*0.022	*0.026	0.596	0.332
Methanobacteriaceae Methanobrevibacter	*0.043	*0.004	0.857	0.934	*0.007	0.113	0.476	*0.018	0.068	0.165	0.389
Methanobacteriaceae Methanobrevibacter	*0.030	*0.026	0.221	*0.022	*0.002	*0.010	0.114	0.112	0.460	0.715	0.996
Ruminococcaceae Ruminococcus	0.125	0.108	0.084	*0.022	*0.048	*0.028	1.000	0.733	0.372	0.470	*0.043
Christensenellaceae Christensenellaceae R-7 group	0.124	0.496	*0.010	0.098	0.090	*0.035	0.157	0.553	0.318	*0.030	*0.049
Christensenellaceae Christensenellaceae R-7 group	*0.035	0.139	*0.046	0.217	0.188	0.180	0.242	0.790	0.113	*0.002	*0.004

*P values > 0.05 were considered significant.

Table S6. P values of the Spearman correlations between health parameters and the vaginal microbiome of Holstein cows at calving time.

Taxa classification family - genus	*P values*											
	NEFA	BHB	Iron	Total protein	Albumin	Triglycerides	Cholesterol	Hp	Lactation number	Milk production	BSC	Weight
Enterobacteriaceae Escherichia-Shigella	0.551	0.680	0.418	*0.000	*0.013	0.152	*0.048	0.961	0.807	*0.024	0.133	0.216
Peptostreptococcaceae Romboutsia	0.738	0.554	0.920	*0.047	0.599	*0.028	*0.037	0.273	0.562	0.697	0.487	0.321
Methanobacteriaceae Methanobrevibacter	0.370	*0.045	0.656	*0.014	0.608	0.801	0.389	0.736	*0.031	0.801	0.505	0.315
Lachnospiraceae Roseburia	*0.017	*0.005	0.378	0.302	0.941	0.572	0.963	0.914	*0.013	0.495	0.654	0.441
Christensenellaceae Christensenellaceae R-7 group	*0.015	*0.006	0.387	0.397	0.892	0.561	0.926	0.877	*0.013	0.588	0.486	0.348
Pseudomonadaceae Pseudomonas	0.955	0.289	*0.028	*0.010	*0.010	*0.011	*0.011	0.569	0.158	0.898	0.846	0.619

*P values > 0.05 were considered significant.

Figure S1. Box and whisker plots representing the median, minimum and maximum values of score adopted to realize the mammary gland and colostrum examination immediately after calving.

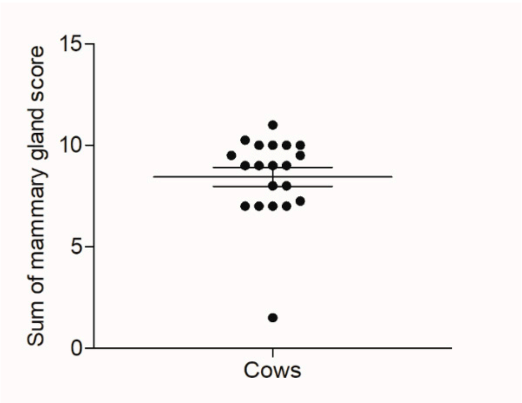

Figure S2. Box and whisker plots representing the median, minimum and maximum values of mammary gland and colostrum health from Holstein cows at the time of calving. SCC: Somatic cells count.

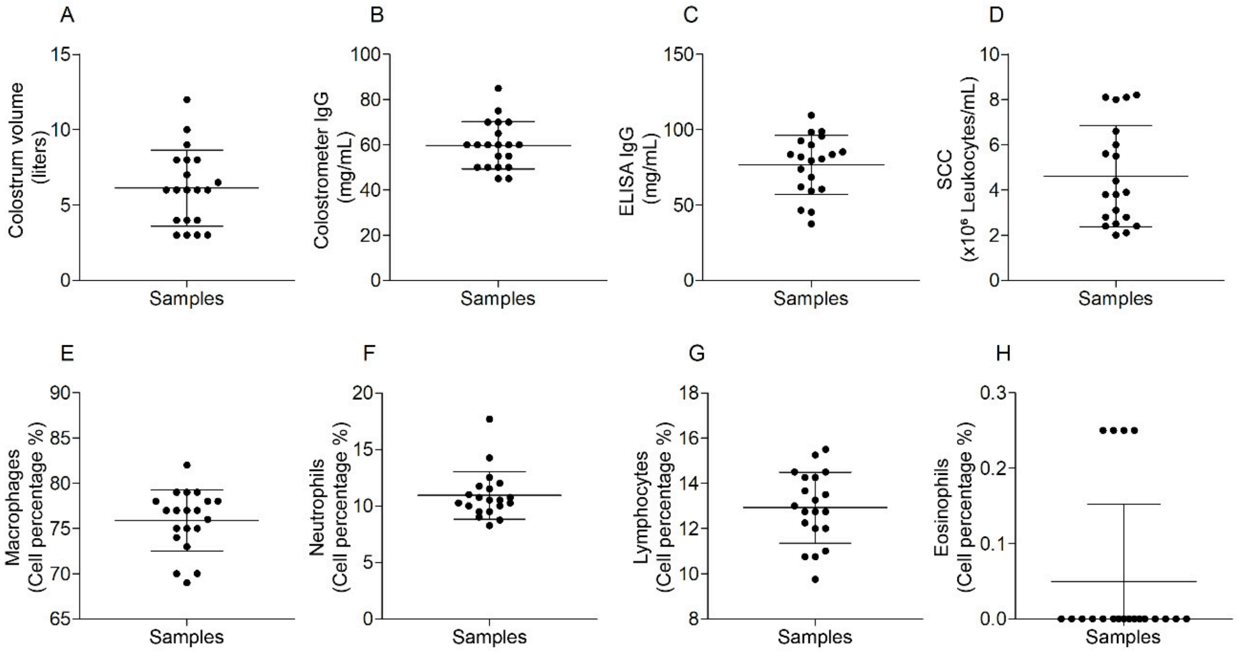

Figure S3. Box and whisker plots representing the median, minimum and maximum values of metabolic, protein and inflammatory parameters from Holstein cows at the time of calving. BSC: Body score corporal; NEFA: non-esterified fatty acids ¸ BHB: β-hydroxybutyrate.

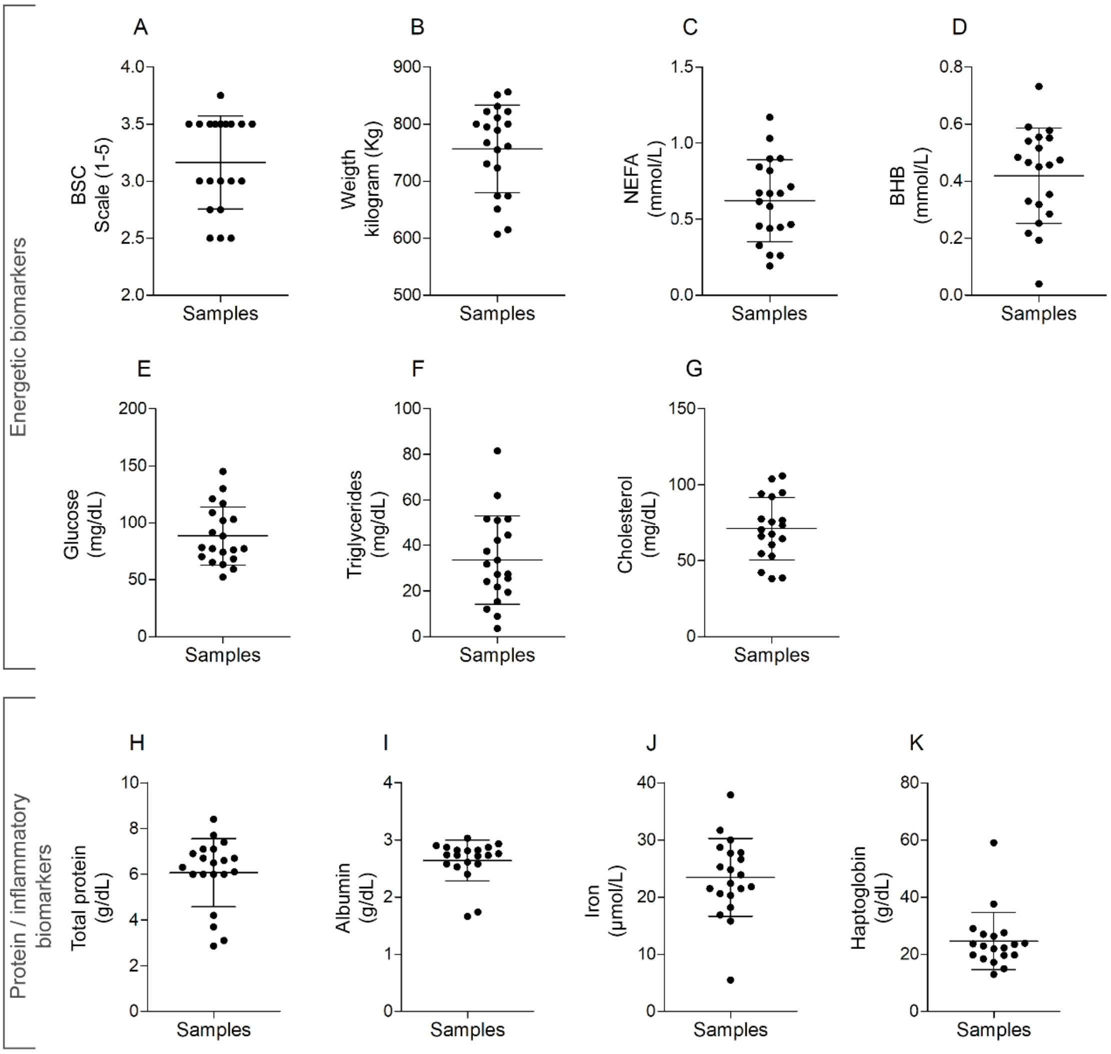

Supplement: Supplementary file 1 [file animals-12-00003-s001.zip › animals-1447011-supplementary.pdf]
